# Causal relationship between immune cells and hepatocellular carcinoma: a Mendelian randomisation study

**DOI:** 10.7150/jca.96744

**Published:** 2024-06-03

**Authors:** Jingyi Tang, Shengke Zhang, Lai Jiang, Jie Liu, Jiayu Xu, Chenglu Jiang, Zipei Chen, Xuancheng Zhou, Claire Fuller, Jinbang Huang, Haiqing Chen, Guanhu Yang, Changsong Bai, Defeng Yin, Bo Li, Hao Chi

**Affiliations:** 1 Department of General Surgery (Hepatopancreatobiliary surgery), The Affiliated Hospital, Southwest Medical University, Luzhou 646000, China.; 2 Academician (Expert) Workstation of Sichuan Province, Metabolic Hepatobiliary and Pancreatic Diseases Key Laboratory of Luzhou City, The Affiliated Hospital, Southwest Medical University, Sichuan, China.; 3 Department of Clinical Medicine, Southwest Medical University, Luzhou 646000, China.; 4 Department of General Surgery, Dazhou Central Hospital, Dazhou 635000, China.; 5 School of Science, Minzu University of China, Beijing, 100081 China.; 6 Department of Chemical and Biomolecular Engineering, Whiting School of Engineering, Johns Hopkins University, Baltimore, Maryland, USA.; 7 Department of Specialty Medicine, Ohio University, Athens 45701, OH, USA.; 8 Department of General Surgery, Xuyong People's Hospital, Luzhou, China.; 9 Department of Emergency Medicine, The Affiliated Hospital, Southwest Medical University, 646000 Luzhou, China.; 10 Department of Emergency Medicine, Xuyong People's Hospital, Luzhou, China.

**Keywords:** HCC, 731 immune traits, Mendelian Randomization, Single-cell RNA sequence, Pseudo-time analysis, Tumor microenvironment

## Abstract

**Background:** Hepatocellular carcinoma (HCC), the predominant malignancy of the digestive tract, ranks as the third most common cause of cancer-related mortality globally, significantly impeding human health and lifespan. Emerging immunotherapeutic approaches have ignited fresh optimism for patient outcomes. This investigation probes the link between 731 immune cell phenotypes and HCC through Mendelian Randomization and single-cell sequencing, aiming to unearth viable drug targets and dissect HCC's etiology.

**Methods:** We conducted an exhaustive two-sample Mendelian Randomization analysis to ascertain the causal links between immune cell features and HCC, utilizing publicly accessible genetic datasets to explore the causal connections of 731 immune cell traits with HCC susceptibility. The integrity, diversity, and potential horizontal pleiotropy of these findings were rigorously assessed through extensive sensitivity analyses. Furthermore, single-cell sequencing was employed to penetrate the pathogenic underpinnings of HCC.

**Results:** Establishing a significance threshold of pval_Inverse.variance.weighted at 0.05, our study pinpointed five immune characteristics potentially elevating HCC risk: B cell % CD3- lymphocyte (TBNK panel), CD25 on IgD+ (B cell panel), HVEM on TD CD4+ (Maturation stages of T cell panel), CD14 on CD14+ CD16- monocyte (Monocyte panel), CD4 on CD39+ activated Treg ( Treg panel). Conversely, various cellular phenotypes tied to BAFF-R expression emerged as protective elements. Single-cell sequencing unveiled profound immune cell phenotype interactions, highlighting marked disparities in cell communication and metabolic activities.

**Conclusion:** Leveraging MR and scRNA-seq techniques, our study elucidates potential associations between 731 immune cell phenotypes and HCC, offering a window into the molecular interplays among cellular phenotypes, and addressing the limitations of mono-antibody therapeutic targets.

## 1. Introduction

HCC as the predominant form of liver cancer and a leading malignant entity within the digestive system, is recognized for its substantial contribution to global cancer mortality, securing its position as the third most common cause of cancer-related deaths 1-3. The escalating incidence and mortality rates associated with HCC signal a significant challenge to public health and longevity 4, 5. Contemporary therapeutic strategies for HCC are transitioning from conventional surgical interventions, such as liver transplants, resections, percutaneous ablations, and radiotherapy, towards a more varied immunotherapeutic approach 6-9. The advent of molecular targeting agents, including kinase and angiogenesis inhibitors, alongside immune checkpoint inhibitors, introduces a new spectrum of hope for patients battling this condition 10.

The liver's role in fostering immune tolerance is paradoxically juxtaposed against its susceptibility to autoimmune conditions, underscoring a complex interplay of genetic regulation within immune cells that selectively influences the risk of autoimmune diseases at the cellular subtype level 11. This nuanced understanding aids in pinpointing precise drug targeting pathways and fosters the development of targeted treatment strategies for autoimmune disorders 12. Our investigation into the relationship between 731 immune cell phenotypes and HCC aims to provide a foundation for identifying potent drug targets and deepening the comprehension of HCC's pathogenesis.

Utilizing genetic variations as proxies for exposure levels, Mendelian Randomization (MR) offers a robust framework for establishing causality within exposure-outcome dynamics, free from the biases inherent in conventional observational studies 13, 14. Single nucleotide polymorphisms (SNPs) were selected as Instrumental variables (IVs) in the Genome-Wide Association Studies (GWAS) database of exposures and outcomes 15. Grounded in the principles of Mendelian inheritance, MR's causal inference is crucial for the identification and repurposing of therapeutic agents 16. MR has emerged as a powerful investigative tool in HCC research, enabling the elucidation of causal relationships within this field 17. Previous studies employing MR have provided significant insights into the determinants of HCC susceptibility. Notable findings include the identification of potential bidirectional causal associations with comorbidities such as depression 18, as well as the crucial role of hepatic function markers in the complex pathogenesis of HCC 19. Additionally, the identification of specific genetic loci and pathways involved in the etiology of HCC has provided valuable insights into potential therapeutic targets 20. The proficient application of Mendelian randomization analyses serves as a cornerstone in our ongoing understanding of the mechanistic underpinnings of HCC, offering significant promise for preventive strategies and therapeutic interventions.

Our analysis, leveraging a comprehensive two-sample MR approach, seeks to elucidate the causal connections between immune cell attributes and HCC, building on prior observations of their association with liver diseases 12, 21. Moreover, our MR analysis fulfills the following three hypotheses: (1) IV is strongly associated with exposure (731 immune phenotypes), (2) IV is not associated with confounders and there is no direct association with HCC, and (3) IV has an effect on HCC only through exposure 22.

The advent of single-cell RNA sequencing (scRNA-seq) has marked a paradigm shift in genomics, enabling the dissection of gene expression at the individual cell level. This technology illuminates the vast cellular diversity, offering unparalleled insights into cellular heterogeneity 23. By integrating MR findings with scRNA-seq data, our research not only clarifies the association between immune cell traits and HCC but also unravels the intricate regulatory interplay between immune cell phenotypes, genes, and the developmental pathways of cellular lineages 24.

## 2 Materials and Methods

### 2.1 GWAS data for HCC

The FinnGen project, a large-scale genomic initiative, provided the GWAS statistics for HCC, encompassing 287,590 Finnish participants (Cases=453, Controls=287,137). This comprehensive study leverages over half a million Finnish biospecimens, integrating genetic variations with extensive health data to elucidate the genetic underpinnings of disease predisposition and pathophysiology.

### 2.2 GWAS data for immune phenotypes

Publicly accessible GWAS summary statistics for a spectrum of 731 immune phenotypes, ranging from absolute cell counts (AC; n=118), median fluorescence intensity (MFI; n=389) indicative of surface antigen levels, to morphological parameters [MP; n=32] and relative cell counts (RC; n=192), are cataloged (accession numbers GCST0001391 to GCST0002121). These data encapsulate diverse immune cells, including B-cells, CDC, various T-cell maturation stages, monocytes, myeloid cells, TBNK (T-cells, B-cells, natural killer cells), and Treg panels, underpinning the genetic basis of immune response variability 12, 16. Originating from 3,757 European individuals, the dataset is distinguished by its comprehensive analysis and absence of overlapping cohorts, enabling precise heritability estimations within our unique family-based cohort study.

### 2.3 Instrumental variables selection

Mendelian randomization of selected instrumental variables for genetic variation must be based on three fundamental assumptions: the correlation hypothesis, which postulates a strong correlation between SNPs and exposure factors; the independence hypothesis, which asserts that SNPs are not affected by various confounders; and the exclusivity hypothesis, which argues that SNPs affect the outcome only through exposure factors 25, 26. In our study, IVs for each immune phenotype were identified with a stringent significance threshold of 5e-8, employing PLINK (v1.90) for SNP trimming based on linkage disequilibrium (LD; r2 < 0.001 within a 10,000 kb radius). This meticulous selection process was mirrored in our reverse Mendelian Randomization analysis for HCC, culminating in the identification of 427 IVs for subsequent investigation.

### 2.4 MR analysis

Employing inverse variance weighting (IVW) and additional robust methods including Mendelian Randomization-Egger (MR-Egger), weighted median, simple mode, and weighted mode, we scrutinized the causal nexus between 731 immunophenotypes and HCC. Through the application of Cochran's Q statistic for IV heterogeneity assessment, MR-Egger for horizontal pleiotropy exclusion, and visual tools like scatter and funnel plots alongside Leave-one-out analysis, we ensured rigorous evaluation of the causal inferences. Furthermore, MR-PRESSO was instrumental in identifying and amending horizontal pleiotropy outliers, fortifying the integrity of our Mendelian Randomization analysis.

### 2.5 Single-cell RNA data acquisition and processing

To elucidate the complexity of HCC, we performed a meticulous analysis using the GSE235057 dataset obtained from the Gene Expression Omnibus (GEO). Crafted by the diligent efforts of Chen et al., this dataset comprises a compelling juxtaposition: 10 specimens of hepatocellular carcinoma tissue juxtaposed with an equal number of adjacent non-tumor tissue samples. This strategic sampling design not only accentuates the salient attributes inherent to tumorous tissues but also affords a clear contrast against the backdrop of normal physiological counterparts. Consequently, this discerning approach serves to mitigate potential confounding variables stemming from biological heterogeneity, thereby elevating the precision and fidelity of our investigative endeavors. This initial selection was followed by an integrative analysis, culminating in a single-cell RNA sequencing (scRNA-seq) dataset of 86,163 cells, complete with gene expression and phenotype matrices. Subsequent to rigorous quality control and normalization procedures, we identified the top 2,000 variable genes, employing the Seurat package's FindVariableFeatures function for gene expression normalization across individual cells.

### 2.6 Cell annotation and visualization

Determining the number of principal components (PCs) through the application of the ScaleData and RunPCA functions, we opted for PC=10. Dimensionality reduction was then achieved using t-Distributed Stochastic Neighbor Embedding (t-SNE) and Uniform Manifold Approximation and Projection (UMAP), allowing for a concise representation of the principal components. Cell populations within the tumor microenvironment (TME) were annotated and visualized leveraging cell marker genes via the Identities and DimPlot functions. Additionally, the distribution of various cell types within HCC and adjacent tissues was depicted using bar graphs.

### 2.7 Pseudo-temporal trajectory inference

Utilizing the Monocle3 R package, we embarked on trajectory inference for assorted cell types within HCC specimens. This process integrated dimensionality reduction clustering via UMAP and PCA, followed by the amalgamation of Monocle and Seurat objects to enhance trajectory analysis. The Learn_graph function facilitated the inference of cell trajectories, establishing a visual representation of cellular developmental pathways. Root cells were pinpointed using the Order_cells function and existing biological insights, enabling the depiction of cellular developmental trajectories. Furthermore, the expression patterns of the top 10 differentially expressed genes, ordered by the Moran index, were simulated to illuminate the cellular evolution within HCC.

### 2.8 Constructing cellular communication networks

Employing the CellChat R package (version 1.6.1) in conjunction with Seurat object-derived gene expression data, we simulated intercellular communication based on the human database. The NetVisual function was instrumental in illustrating the varied facets of cellular communication networks, shedding light on the intricate signaling pathways and ligand-receptor pairs across cell clusters.

### 2.9 Gene Set Enrichment Analysis

Differential gene expression across cell subtypes was ascertained using the FindMarkers function, setting the stage for Gene Set Enrichment Analysis (GSEA). By pre-ranking all expressed genes and employing the clusterProfiler package, we determined the normalized enrichment scores (NES) and false discovery rate (FDR) q-values, further elucidating specific KEGG signaling pathways through targeted visualization.

### 2.10 Statistical analysis

Comprehensive statistical analyses were conducted using R software (version 4.3.1), with a significance threshold set at two-sided p-values < 0.05. The Student's t-test was applied to normally distributed variables, while the Wilcoxon test was utilized for those not normally distributed, facilitating comparative analyses between groups.

## 3. Results

### 3.1 Association of immune traits with HCC risk

In our quest to comprehensively identify immune traits that potentially exhibit a positive association with HCC, we opted against applying corrections for multiple testing 27, 28 in our subsequent exploratory analyses. Our primary analytical tool was the IVW method, augmented by other methods to conduct two-sample MR analyses.

**Figure 1:**
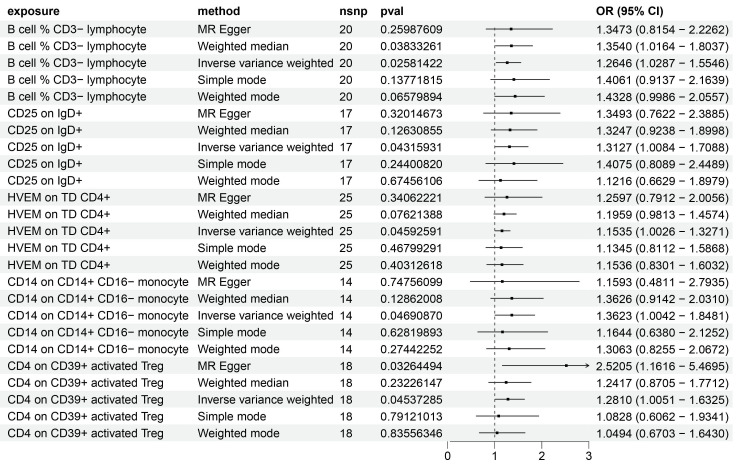
Forest plot showing the risk of 5 risk factors for HCC.

Utilizing the IVW method and setting the significance threshold at p<0.05, we identified five immune traits that may act as risk factors for HCC (Figure 1). Notably, the B cell % CD3- lymphocyte (TBNK panel) ratio's association with HCC risk yielded an odds ratio (OR) of 1.3473 (95% CI=0.8154-2.2262, p=0.02581422). This finding was corroborated by analyses using the weighted median (OR=1.3540, 95% CI=1.0164-1.8037, p=0.03833261) and weighted mode (OR=1.4328, 95% CI=0.9986-2.0557, p=0.06579894) methods. The OR for CD25 on IgD+ (B cell panel) estimated via the IVW method was 1.3127 (95% CI=1.0084-1.7088, p=0.04315931), with consistent β-value directions across all analysis methods. The HVEM on TD CD4+ (Maturation stages of T cell panel) exhibited an OR of 1.1535 (95% CI=1.0026-1.3271, p=0.04592591), with consistent β-value directions noted. Similarly, for CD14 on CD14+ CD16- monocyte (Monocyte panel) and CD4 on CD39+ activated Treg (Treg panel), the IVW method estimated ORs of 1.3623 (95% CI=1.0042-1.8481, p=0.04690870) and 1.2810 (95% CI=1.0051-1.6325, p=0.04537285), respectively, with consistent β-value directions. Additionally, the MR Egger analysis indicated a significant OR of 2.5205 (95% CI=1.1616-5.4695).

Regarding protective factors against HCC, a noteworthy observation was the strong association of 16 out of 30 immune cell phenotypes with BAFF-R expression (Supplementary Table 1), alongside a notable OR for CD4 on CD39+ resting Treg of 0.62 (95% CI=0.43-0.88, p=0.008433174), highlighting CD4 on CD39+ activated Treg as a risk factor.

Further, the robustness of our associations was validated through multiple methodologies (Supplementary Table 1), with heterogeneity and pleiotropy analyses (Q test, Egger intercept, MR-PRESSO) indicating negligible heterogeneity and horizontal pleiotropy among the SNPs associated with the five immune traits. Sensitivity analyses, including MR leave-one-out and weighted ratio MR, confirmed the stability of our findings (Supplementary Figures 1-4). Notably, MR-PRESSO's final test underscored the isotropy of causal estimates to beta effect values, enhancing the confidence in our positive results (Supplementary Table 2).

Additionally, employing reverse MR analysis, we examined the potential influence of HCC on these immune traits (Supplementary Table 3), finding no significant reverse causality, which underscores the potential role of these immune traits as significant contributors to the development and progression of HCC.

### 3.2 scRNA sequencing reveals potential mechanisms

**Figure 2:**
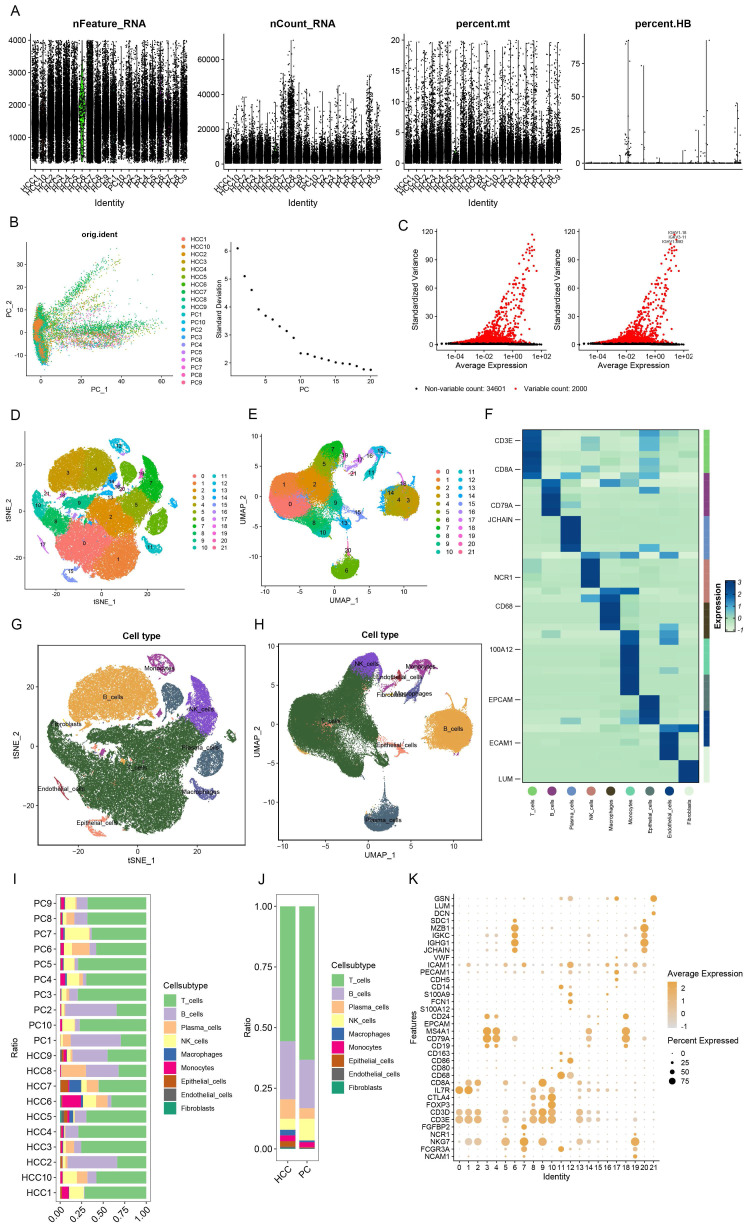
scRNA sequencing reveals potential mechanisms. (A) Single-cell QC. Single cell gene count is between 200-4000, nCount_RNA is 200 or more, and mitochondrial percentage is below 20%. (B) PCA plots indicate a small batch effect; Elbow plots show that clustering is more reasonable at PC=10. (C) The top 2000 highly variable genes were selected for scaling. (D-E) TSNE and UMAP plots show the initial clustering results:a total of 22 clusters were classified.(F) Heatmaps show the specific cell marker genes used for cellular annotation. (G-H) TSNE and UMAP plots showing 9 cell clusters: T cells, B cells, Plasma cells, NK cells, Macrophages, Monocytes, Epithelial cells, Endothelial cells, Fibroblasts. (I) Bar graphs demonstrates the percentage of cells in HCC and PC tissues. (J) Bar graph demonstrating cell occupancy in combined HCC and PC samples. (K) Bubble graph demonstrating the expression of cell marker genes in each cell cluster.

To meticulously elucidate the nexus between immune traits and HCC, we capitalized on single-cell RNA (scRNA) sequencing data for empirical validation. Stringent preliminary quality control measures were enacted, ensuring that individual cells harbored 200 to 4000 genes with mitochondrial content maintained below 20% (Figure 2A). The foremost 2000 genes, characterized by their high variability, were selected for normalization (Figure 2C), and the analysis of tumor versus normal samples from an identical dataset exhibited minimal batch effects (Figure 2B). Utilizing the Elbow plot, we determined PC=10 as optimal for dimensionality reduction, subsequently employing TSNE and UMAP for the depiction of initial clustering outcomes (Figure 2D, E). This stratification yielded 22 distinct clusters, further delineated into nine cell types as per specific marker genes (Figure 2F, K): T cells, B cells, Plasma cells, NK cells, Macrophages, Monocytes, Epithelial cells, Endothelial cells, and Fibroblasts (Figure 2G, H), with the differential cell composition between HCC and control samples graphically represented (Figure 2I,J). Notably, a significant diminution in T cell prevalence was observed in HCC samples, contrary to the marked increase in B and Plasma cells, hinting at the divergent roles immune cells may assume across varying HCC stages. While scRNA sequencing offers a snapshot of cellular dynamics, it is constrained by a limited capacity to thoroughly interrogate the temporal dynamics of cellular functions 29.

### 3.3 Dynamic changes of T cell subpopulations in HCC and study of immune pathways

**Figure 3:**
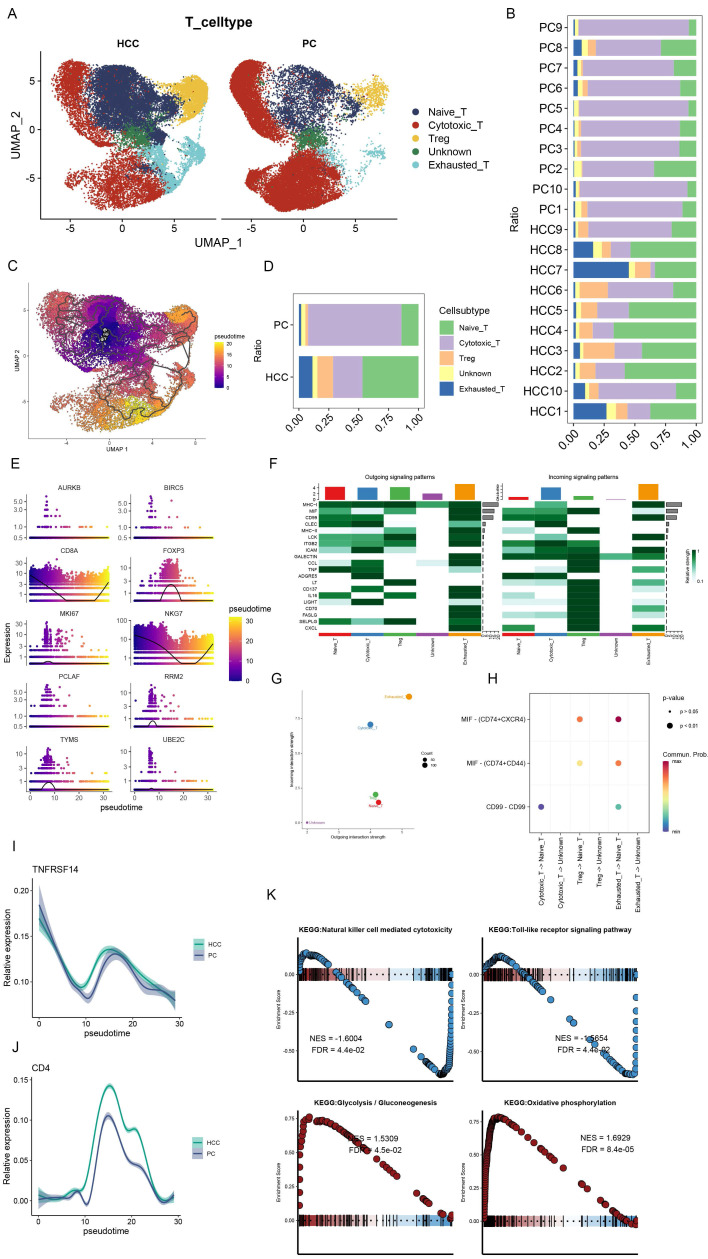
Dynamic changes of T cell subsets in HCC and immune pathway studies. (A) UMAP plot showing the difference in the distribution of T cell subpopulations in HCC and PC, which were categorized into Naive T cells, Cytotoxic T cells, Treg, Unknown, and Exhausted T cells. (B) Bar graph demonstrating the percentage of T cell subpopulations in the tissues of HCC and PC. (C) UMAP plot presenting the developmental trajectory tree of T cell subpopulations. T cells are differentiated into various subpopulations starting with Naive T cells. (D) Bar graph presenting the percentage of T cell subpopulations in the combined HCC and PC samples. The percentage of Cytotoxic T cells in HCC was significantly decreased, while Naive T cells, Treg, and Exhausted T cells were significantly increased. (E) Proposed temporal changes of the top 10 genes ranked by the Moran Index with cell development. (F) Network heatmap showing the signaling pathways of T cell subpopulations. (G) Input versus output signal intensity. Output signals are dominated by Exhausted T cells, whereas output signals are dominated by Treg emitted (H) Bubble diagram showing differences in signaling intensity of specific pathways (I) Differences in changes of TNFRSF14 (HVEM) in PC and HCC during the course of following cell development. (J) Differences in CD4 changes in PC and HCC during development with cells. (K) Single-cell GSEA. natural killer cell mediated cytotoxicity, Toll-like receptor signaling pathway downregulation; Glycolysis -Gluconeogenesis and Oxidative phosphorylation were upregulated in T cell subsets.

In a subsequent exploration of T cell heterogeneity within HCC, we delineated five distinct T cell subsets: Naïve T cells, Cytotoxic T cells, T regulatory (Treg) cells, an Unknown category, and Exhausted T cells (Figure 3A). It is noteworthy that the proportion of Cytotoxic T cells was substantially reduced in HCC, in contrast to the augmented presence of Naïve T cells, Treg, and Exhausted T cells (Figure 3B, D). Following the excision of tumor tissues, pseudo-temporal analyses were conducted, with the UMAP plots illustrating the developmental trajectory of T cell subsets, thereby validating the accuracy of our cell annotations (Figure 3C). This differentiation trajectory commences with Naïve T cells evolving into diverse subsets, aligning with established biological insights. Further, we investigated the temporal dynamics of the top 10 ranked genes by the Moran index in relation to cell development (Figure 3E), revealing significant fluctuations in CD8A, FOXP3, and NKG7. Specifically, CD8A and NKG7 exhibited a decrease followed by an increase, whereas FOXP3 manifested the highest expression midway through the sequence. These genes, pivotal for annotating T-cell subsets, mirror the developmental trajectory of T-cell subtypes, indirectly reflecting shifts in T-cell subset composition during HCC progression. The interplay between cytotoxic and regulatory T cells within tumors is strongly associated with recurrence and overall survival in HCC, underscoring the prognostic potential of cellular markers 30. Furthermore, the differential expression of TNFRSF14 (HVEM) and CD4 in HCC and control samples was meticulously analyzed, revealing a marginally higher expression of TNFRSF14 in HCC (Figure 3I). RT-qPCR analyses corroborated elevated HVEM levels in HCC tissues relative to adjacent non-tumor tissues 31, with CD4 expression peaking midway through the temporal series, mirroring FOXP3 dynamics (Figure 3J).

The network heatmap unveiled the signaling cascades within T cell subsets, with Exhausted T cells and Treg cells predominantly modulating output signals (Figure 3F, G). Intriguingly, the MIF signaling pathway was found to be hyperactivated in both Treg and Exhausted T cells (Figure 3H). Wang et al. documented elevated serum and tissue MIF levels in HCC patients compared to healthy controls and adjacent non-tumor liver tissues, respectively 32. GSEA enrichment analysis highlighted the downregulation of Natural killer cell-mediated cytotoxicity and Toll-like receptor signaling pathways, alongside the upregulation of Glycolysis-Gluconeogenesis and Oxidative phosphorylation pathways within T cell subsets (Figure 3K), suggesting a metabolic adaptability and lack of suppression in Treg cells within the HCC milieu. This metabolic versatility supports Treg cell-mediated immunosuppression, facilitating tumor immune evasion 33, 34.

### 3.4 Monocyte signaling pathway activity and metabolic activity

**Figure 4:**
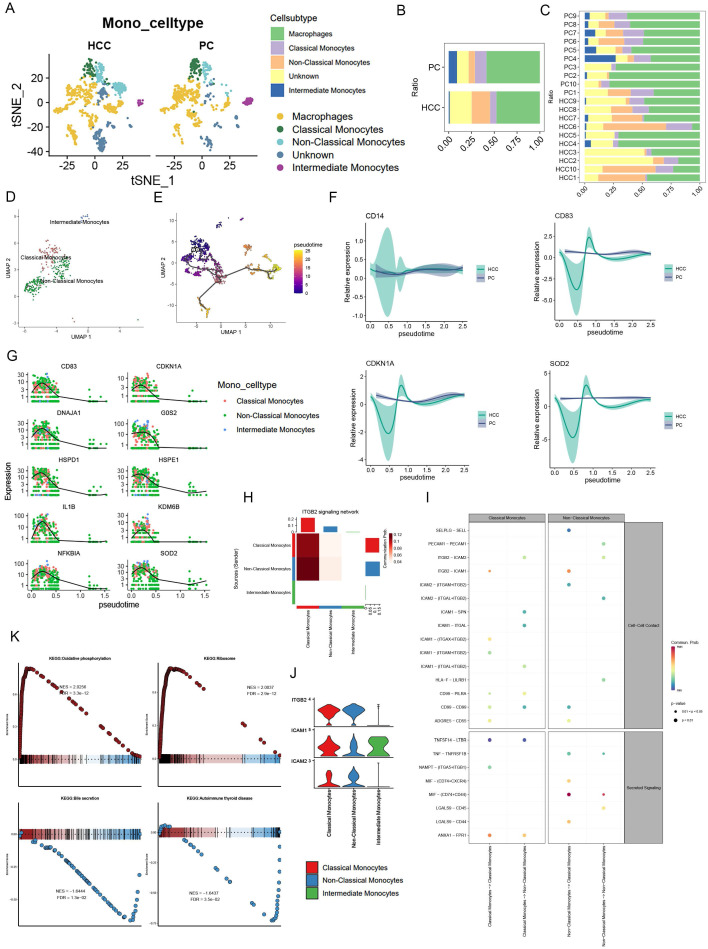
Monocyte signaling pathway activity and metabolic activity. (A) UMAP plot showing the difference in the distribution of monocyte subpopulations in HCC versus PC, except for Unknown and Macrophages, where monocytes were further categorized into Classical Monocytes, Non-Classical Monocytes, and Intermediate Monocytes. (B) Bar graph demonstrating the percentage of monocyte subpopulations in the combined HCC and PC samples. (C) Bar graph demonstrating the percentage of monocyte subpopulations of HCC and PC sample tissues. (D-E) UMAP plots presenting the developmental trajectory tree of monocyte subpopulations. (F) Differences in CD14, CD83, CDDKN1A, COD2 with cell development in HCC and PC. (G) The proposed temporal changes of the top 10 genes ranked by the Moran index with cell development. (H) Heatmap format visualizing the ITGB2 signaling network with Classical Monocytes and Non-Classical Monocytes as the main emitters (I) Bubble diagram showing the signaling pathway between monocyte subpopulations, Non-Classical Monocytes were found to be active in the MIF pathway (J) Violin graph demonstrates the receptor expression of the ITGB2 signaling pathway, and only Intermediate Monocytes specifically express ICAM1.(K) Monocyte GSEA: Oxidative phosphorylation, Ribosome expression is upregulated; while Bile secretion, Autoimmune thyroid disease were downregulated.

Following initial annotation, we meticulously isolated monocytes, proceeding with dimensionality reduction clustering. This categorization unveiled Classical Monocytes, Non-Classical Monocytes, and Intermediate Monocytes, alongside Unknown and Macrophages, with Non-Classical Monocytes emerging as predominantly associated with HCC, in contrast to the lesser extents of the other types (Figure 4A, B, C). Subsequent pseudotemporal analysis delineated Non-Classical Monocytes' progressive differentiation (Figure 4D, E). Intriguingly, analysis via Moran's index revealed a gene expression pattern that surged before tapering off as cellular development advanced (Figure 4G). In comparing HCC with precursor cells, CD14 alterations were negligible, whereas CD83, CDKN1A, and COD2 exhibited more pronounced fluctuations in HCC, suggesting differential regulatory mechanisms (Figure 4F). Signaling pathway analyses, particularly through bubble plots, highlighted Non-Classical Monocytes' significant activity within the MIF pathway, with Intermediate Monocytes displaying only subdued signals (Figure 4I). Furthermore, the ITGB2 signaling network, illustrated via heatmap, pinpointed Classical and Non-Classical Monocytes as pivotal communicators, as evidenced by a violin plot showcasing pathway-specific receptor expression, with ICAM1 uniquely expressed by Intermediate Monocytes (Figure 4H, J).

Exploration into monocytes' KEGG pathways revealed upregulation in oxidative phosphorylation and ribosome expression, contrasting with downregulation in bile secretion and autoimmune thyroid disease pathways (Figure 4K). It's noteworthy that HCC-derived exosome PKM2 fosters metabolic reprogramming in monocytes, leading to phosphorylated STAT3 and M2 polarization in macrophages, thereby facilitating a metabolic shift towards aerobic glycolysis in tumor-associated monocytes and macrophages 35-37. This delineation of metabolic pathways underscores the distinct roles of classical and non-classical monocytes, with the former enhancing glycolytic pathways and the latter favoring oxidative phosphorylation, thereby emphasizing the pivotal oxidative phosphorylation role of non-classical monocytes.

## 4. Discussion

Leveraging a comprehensive repository of genetic and single-cell sequencing data, our investigation rigorously elucidated the causal interplay between 731 immune cell phenotypes and HCC. Employing state-of-the-art single-cell methodologies allowed for an in-depth exploration of immune cell mechanisms within HCC contexts. Our findings pinpointed five immune traits with definitive causal links to HCC, highlighting several BAFF-R-associated immune phenotypes as protective against the condition. Further examination at the single-cell level shed light on these immunophenotypes.

The B cell % CD3- lymphocyte (TBNK panel) metric, indicative of B cells lacking CD3 within a lymphocyte subset (TBNK, comprising T cells, B cells, and natural killer cells), served as a key focus 38. Our analysis demonstrated a correlation between increased B cell % CD3- lymphocytes and elevated HCC risk. This finding gains further traction as scrutinized through single-cell evaluations, which unveil a pronounced surge in B cell compositions within HCC specimens relative to their healthy counterparts. This empirical evidence underscores the pivotal involvement of B cells in the intricate pathogenesis of HCC, thereby setting a sturdy foundation for forthcoming inquiries into the intricate web of B cell-mediated mechanisms propelling HCC progression.

CD25 on IgD+ (B cell panel) describes a specific subpopulation of CD25 (IL-2Rα) on the surface of B cells, and this subpopulation is characterized by its expression of immunoglobulin D (IgD) 12. IgD is normally expressed on the surface of mature B cells that are ready to participate in antigen recognition and immune response. When B cells are stimulated and activated, they may undergo further differentiation and subpopulation differentiation to perform specific immune functions 39. CD25 is associated with processes such as cellular immune activation, regulation of T-cell interactions, cell proliferation and survival, and immunomodulation 11, 40. It has been shown that CD25(+) B cells produce higher levels of IL-6, IL-10 and INFgamma in response to different TLR agonists and are more inclined to present cognate antigens to CD4(+) T cells. CD25-expressing B cells spontaneously secrete immunoglobulins of the IgA, IgG and IgM subclasses, and outperform CD25(-) B cells in terms of migratory capacity 40, 41. Intriguingly, our discoveries posit that augmented CD25 expression on IgD+ B cells may harbor an association with heightened hepatocellular carcinoma (HCC) susceptibility. This intriguing nexus between B cell activation status and immune functional modulation suggests a potential avenue for further exhaustive exploration, urging subsequent in-depth investigations.

CD14 on CD14+ CD16- monocyte (Monocyte panel) is an indicator used in immunology or flow cytometry to characterize cell surface markers. The results of the study revealed that as CD14 on CD14+ CD16- monocyte increased, the risk of HCC also tended to increase. Monocytes are usually categorized into three subtypes: Classical Monocytes (CD14++ CD16-), Intermediate Monocytes (CD14+ CD16+) and Non-Classical Monocytes (CD14low CD16+). Our hypothesis posits that alterations in CD14, particularly within Classical Monocytes, may have a profound impact on HCC development. CD14 serves as a co-receptor for toll-like receptor 4 (TLR4) and TLR2, initiating the TLR signaling pathway and prompting the secretion of inflammatory mediators such as reactive oxygen species (ROS), interleukin-1 β (IL-1β), and IL-6^42^, ^43^. Studies have demonstrated that cytokines linked to CD14, including interleukin-6 (IL-6), IL-8, TNF-α, and IL-10, play roles in the pathogenesis of HCV-induced liver disease and in the regulation of fibrosis in patients with chronic hepatitis C (CHC) 44. At the single-cell level, our research uncovered an increase in Non-Classical Monocytes (CD14^low CD16+) within HCC, indicative of a specific cellular development trajectory. Additionally, Non-Classical Monocytes are prevalent in the inflammatory response of chronic diseases such as systemic lupus erythematosus (SLE) 45. This underscores the substantial impact of monocytes on HCC, informed by both statistical and single-cell analyses, given the interconnected nature of tumor development and chronic inflammation 46.

HVEM on Elevated TD CD4+ (T cell maturation panel) reveals a significant association with increased HCC risk. These TD T cells represent the ultimate stage in memory T cell maturation 12. The pivotal role of HVEM, especially through the BTLA/HVEM pathway, in tumor immunity is underscored by its contribution to immune evasion, suppression of T cell activity, and reduction of cytokine secretion by tumor-infiltrating CD4+ T cells, highlighting the pathway's critical role in the tumor microenvironment 47-49. The correlation between elevated HVEM expression and poorer survival outcomes in various cancers, including esophageal squamous cell carcinoma, HCV-associated HCC, and colorectal cancer, underscores its broader relevance across different malignancies. 50, 51. At the single-cell level, the observed increase in regulatory T cells (Tregs) and decrease in cytotoxic T cells underscore T cell regulation's significance in HCC's pathogenesis, further emphasizing the intricate interplay of immune modulation in cancer development. Therefore, targeting the BTLA/HVEM pathway or modulating T cell regulation could represent promising strategies for enhancing anti-tumor immunity and improving clinical outcomes in HCC patients. Further research is warranted to elucidate the precise mechanisms by which HVEM influences T cell function and its role in shaping the tumor microenvironment.

Statistical analyses have elucidated that an increased CD4 on CD39+ activated Treg (Treg panel) is significantly correlated with HCC, positioning it as a contributory risk factor. Tregs, a subset of CD4+ T cells, are recognized for their pivotal role in enforcing immune tolerance and mitigating tissue damage across a spectrum of immune-mediated conditions. Nevertheless, their presence in solid tumors has emerged as a significant barrier to the efficacy of cancer immunotherapies 52, 53. Previous research in the realm of autoimmune diseases has highlighted the beneficial impact of Tregs in dampening inflammatory responses, advocating for their augmented incorporation in therapeutic strategies targeting these conditions 54. Contrarily, in the context of oncology, Tregs assume a detrimental role by facilitating tumor evasion from immune detection 55. Investigations have revealed that Tregs in their activated state are characterized by elevated expressions of CD39, CD73, and TGF-β 56, 57. Collectively, these findings suggest that CD39+ activated Tregs may impair antitumor immunity by secreting immunosuppressive agents, such as adenosine, or by suppressing the functionality of other immune cells. It is worth noting that they can both hinder antitumor immune responses by promoting immune evasion and potentially enhance autoimmune responses by dampening inflammatory reactions. This duality emphasizes the importance of context-specific modulation of Treg activity in cancer therapy and underscores the need for tailored approaches that consider the intricate balance between immune tolerance and antitumor immunity.

Remarkably, CD4 on CD39+ resting Treg confers protection against HCC, indicating that the relationship between antigen expression and disease risk may inversely vary across different cellular subtypes. This observation underscores the limitation of therapeutic strategies that focus on singular antigen targeting. It suggests a more nuanced approach, advocating for the simultaneous targeting of multiple antigens to achieve cell subtype-specificity, thereby enhancing therapeutic efficacy 12. Such insights are pivotal for the refinement of drug efficacy predictions, urging researchers to adopt strategies that involve the co-targeting of two or more proteins. This could be achieved through the use of targeted delivery mechanisms, such as multispecific monoclonal antibodies or small molecule vectors, to direct the therapeutic agents to specific cell types 12, 58.

Our investigation elucidates that BAFF-R constitutes a pivotal marker for the identification of protective factors across diverse immune cell profiles in HCC. It plays a critical role in the activation and regulation of B cell survival and maturation. Moreover, the BAFF-BAFF-R axis is intricately linked with cancer progression, apoptosis, and inflammation 59, 60. Research conducted by Khlaiphuengsin et al. has highlighted a significant reduction in BAFF-R expression within B cells, a phenomenon intimately correlated with the maturation frequency of B cells in individuals afflicted with HCC 61. Additionally, a diminished expression of BAFF-R has been strongly linked to the advancement of HCC, encompassing increases in tumor dimension and the progression to more advanced stages of the cancer 61. Investigations have also demonstrated that BAFF activity can be attenuated through the employment of an antibody that selectively targets BAFF-R, subsequently enhancing the susceptibility of cancer cells to pharmacological interventions 62. In summary, dysregulation of the BAFF-BAFF-R axis may contribute to the development and progression of HCC through mechanisms involving immune evasion, cell survival, and inflammation-mediated tumor growth. Therefore, modulation of the BAFF-BAFF-R axis may be a promising therapeutic strategy for the treatment of HCC, potentially enhancing the efficacy of existing pharmacological interventions.

In our investigation into the intricacies of metabolic pathways and cellular dialogue, we encountered a remarkable observation: both T cells and monocytes exhibited enhanced regulation on the oxidative phosphorylation pathway and demonstrated pronounced activity within the MIF signaling cascade. When examining intercellular communications, it was evident that within the HCC milieu, characterized by elevated oxidative stress, the MIF pathway facilitated a substantially more robust interaction between malignant and immune cells compared to that observed in non-tumorous hepatocytes 63. Prior research has elucidated that MIF exerts multifaceted effects on both tumor cells and the surrounding stroma by mechanisms that include modulation of tumor cell migration and the suppression of immune cell invasion into tumoral regions 64, 65. The suppression of endogenous MIF expression was associated with a deceleration in tumor cell growth, and similarly, the targeted knockdown of MIF led to a reduction in HCC cell proliferation 66. This evidence suggests a potential interconnection between the oxidative phosphorylation pathway and MIF signaling, offering a novel and promising avenue for future research endeavors.

Our investigation into 731 immune cell phenotypes within HCC patients unveiled numerous noteworthy findings. However, it is imperative to acknowledge certain constraints inherent to this study. First, MR analysis relies on three key assumptions, and ensuring that the selected genetic variants are indeed associated with exposure usually requires large-scale GWAS data to support it. Our study, based on a Finnish database, may have some limitations in terms of representativeness, and therefore these observations need to be validated by independent studies with a larger number of patients. Furthermore, given the retrospective nature of this study, it is susceptible to the influence of confounding variables, such as age and gender, which may skew the results. In addition, single-cell sequencing is highly sensitive and technically challenging and may be affected by factors such as technical noise and batch effects, and validation of results using multiple technology platforms would greatly enhance the accuracy and rigor of the study. It is, therefore, essential to undertake further research to not only verify these preliminary observations across diverse HCC etiologies but also to decipher the underlying mechanisms through which specific immune cell phenotypes contribute to the pathogenesis of hepatocellular carcinoma.

## 5. Conclusion

Leveraging Mendelian randomization analysis, we conducted an exhaustive exploration of the potential links between 731 immune cell phenotypes and HCC. Our findings suggest that five specific immune cell phenotypes could serve as risk factors for HCC, whereas several phenotypes associated with BAFF-R expression might offer protective effects. The nuanced roles of CD4 expression in CD39+ activated versus resting regulatory T cells (Tregs) in HCC progression underscore the limitations of monotherapy approaches targeting a single antibody. Employing single-cell sequencing data, we delved deeper into the molecular underpinnings by which relevant immune cells influence HCC development. The innovative application of a time-sequencing methodology illuminated gene expression alterations linked to the five implicated immune cell phenotypes, laying the groundwork for future research directions.

## Supplementary Material

Supplementary figures and tables.

## Abbreviations

HCC: Hepatocellular Carcinoma
MR: Mendelian Randomization
SNPs: Single Nucleotide Polymorphisms
IVs: Instrumental Variables
GWAS: Genome-Wide Association Studies
scRNA-seq: Single-cell RNA sequencing
AC: absolute cell counts
RC: relative cell counts
LD: linkage disequilibrium
IVW: inverse variance weighting
MR-Egger: Mendelian Randomization-Egger
GEO: Gene Expression Omnibus
scRNA-seq: single-cell RNA sequencing
PCs: principal components
t-SNE: t-Distributed Stochastic Neighbor Embedding
UMAP: Uniform Manifold Approximation and Projection
TME: tumor microenvironment
OR: odds ratio
scRNA: single-cell RNA
CHC: chronic hepatitis C
SLE: systemic lupus erythematosus
