# Supplementary material for: Causal relationship between immune cells and hepatocellular carcinoma: a Mendelian randomisation study
Source: J Cancer. 2024 Jun 3;15(13):4219–31. doi: 10.7150/jca.96744 (PMC11212088; doi:10.7150/jca.96744)
Supplement: Supplementary file 1 — Supplementary figures and tables. [file jcav15p4219s1.zip › supplementary figure legends.docx]

**Supplementary Figure 1:** MR Test. A-E indicate are the results of MR analysis between immune cell phenotype and HCC, respectively. Each point represents a line on each point of the instrumental variable SNP; the horizontal coordinate is the effect of SNP on immune cell phenotype; the vertical coordinate is the effect of SNP on HCC.

**Supplementary Figure 2:** Leave-one-out sensitivity analysis. Shows that the results remain unchanged (ALL>0) when the IVW analysis is rerun after removing a SNP.

**Supplementary Figure 3:** Forest plot: indicates the results of waid ratio mr analysis for a single SNP.

**Supplementary Figure 4:** Funnel plot: shows that there are a few outliers for each of the five immune traits.

**Supplementary Table 1:** Results of causal effects of immune cells on HCC

**Supplementary Table 2:** MR-PRESSO results of causal effects of immune cells on HCC

**Supplementary Table 3:** Causal effects of HCC on immune cells
