# Supplementary figures and images for: Causal relationship between immune cells and hepatocellular carcinoma: a Mendelian randomisation study
Source: J Cancer. 2024 Jun 3;15(13):4219–31. doi: 10.7150/jca.96744 (PMC11212088; doi:10.7150/jca.96744)

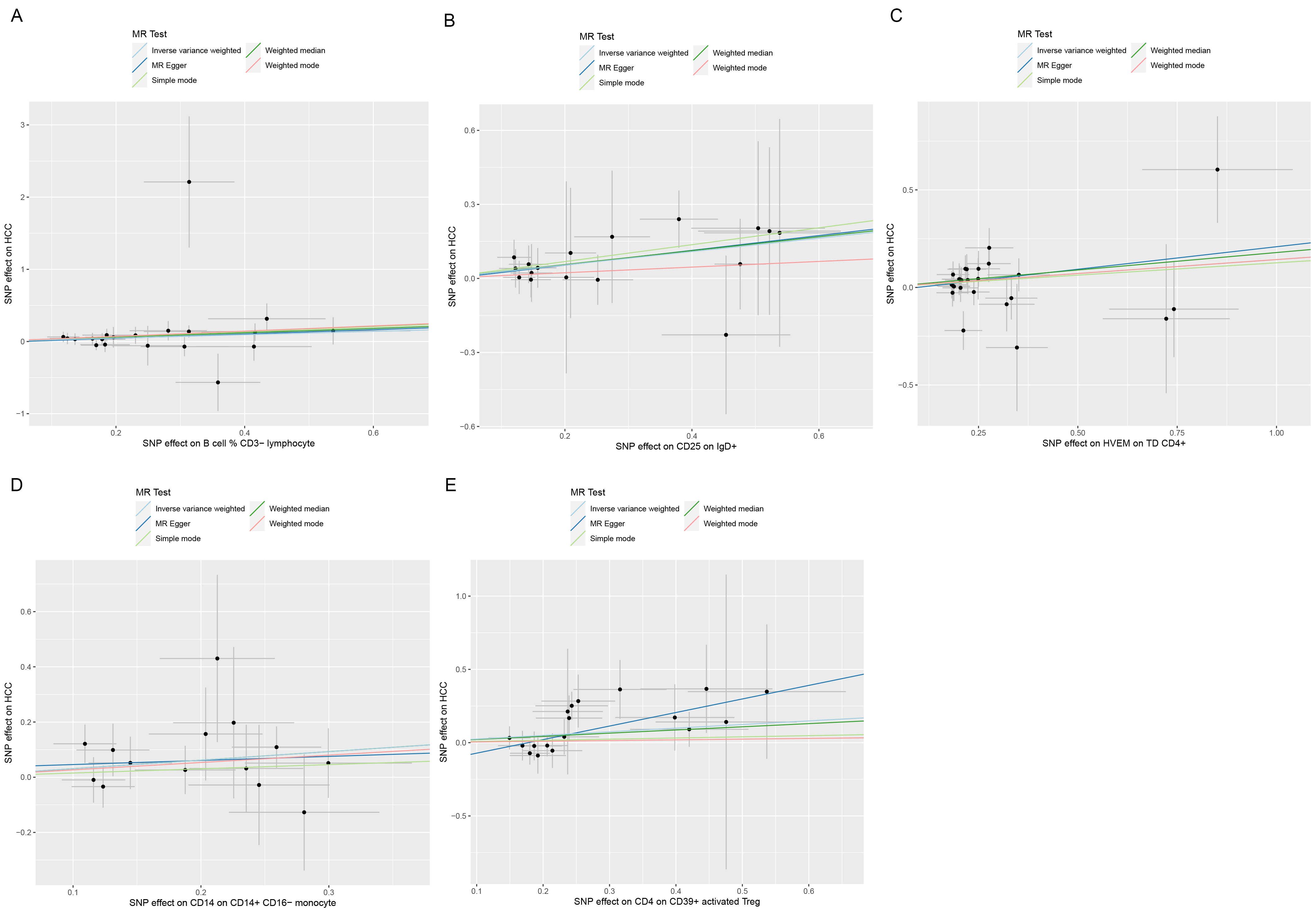

Supplement: Supplementary file 1 — Supplementary figures and tables. [file jcav15p4219s1.zip › Supplementary Figure 1.tif]

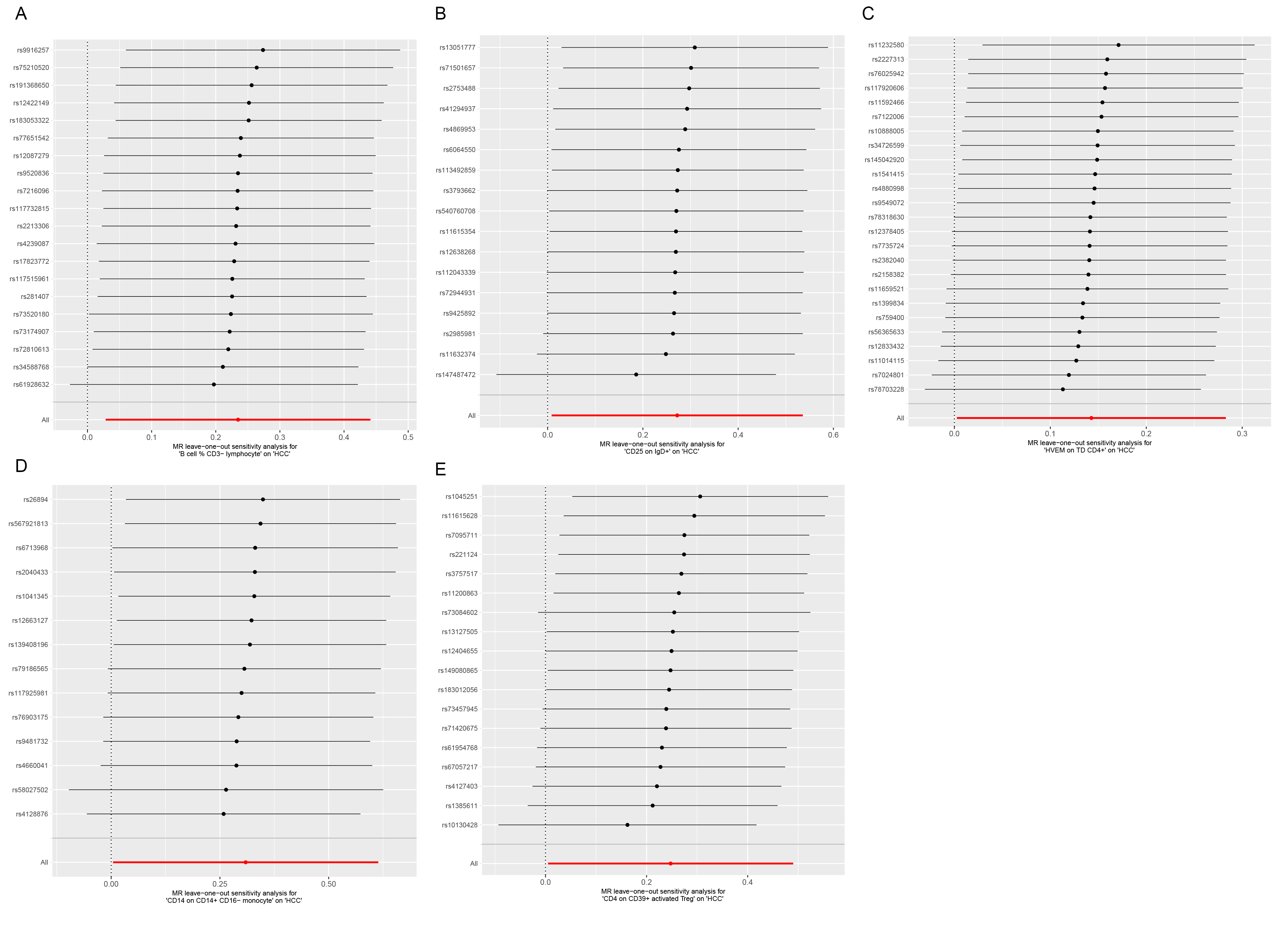

Supplement: Supplementary file 1 — Supplementary figures and tables. [file jcav15p4219s1.zip › Supplementary Figure 2.tif]

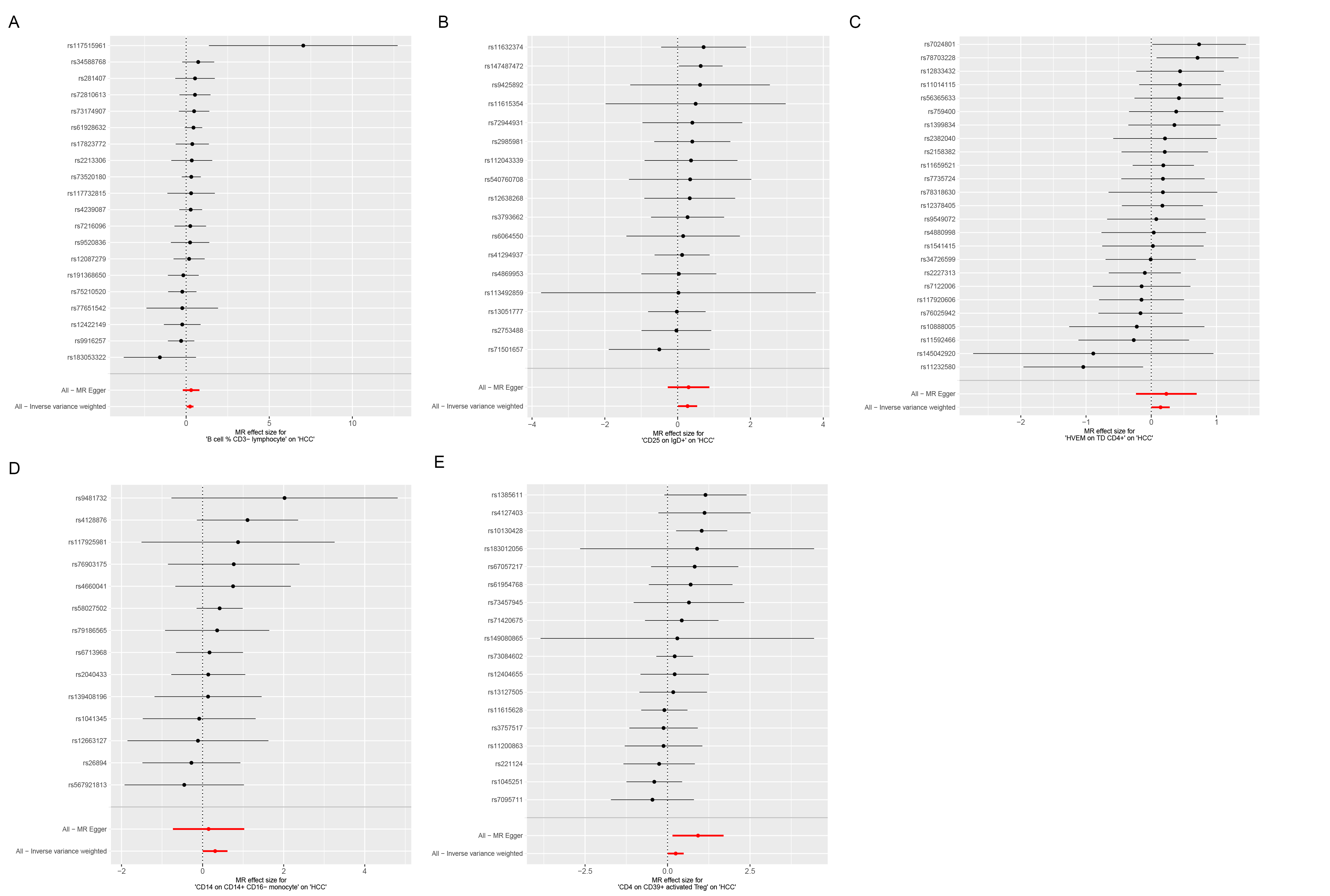

Supplement: Supplementary file 1 — Supplementary figures and tables. [file jcav15p4219s1.zip › Supplementary Figure 3.tif]

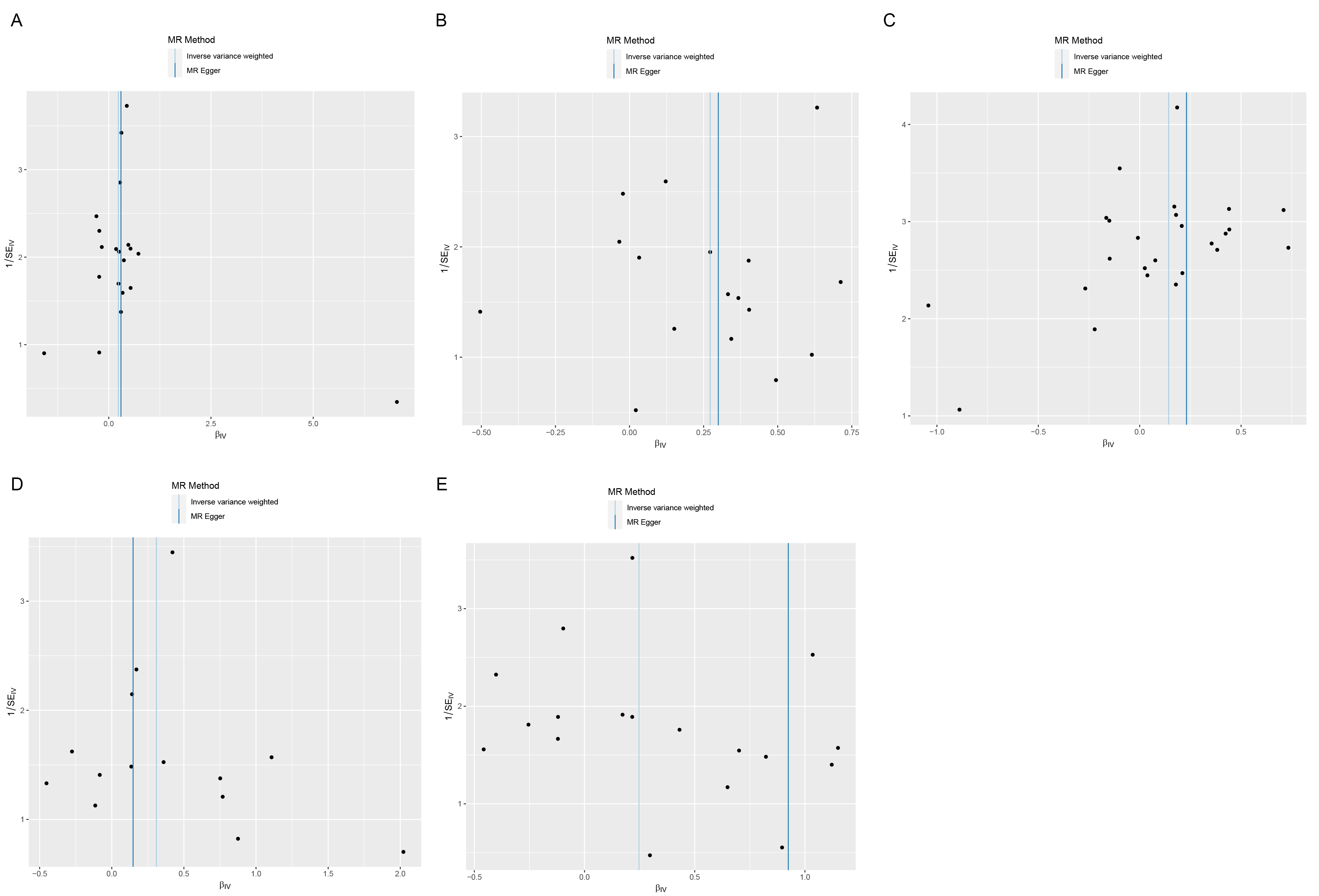

Supplement: Supplementary file 1 — Supplementary figures and tables. [file jcav15p4219s1.zip › Supplementary Figure 4.tif]
